# Critical Examination of the Evidence, Arguments, Verdict, &c

**Published:** 1829-01-01

**Authors:** 


### COMMENTARY.

The Profession is now, at length, tasting the *bitter* drainings of that poisoned chalice, from which it so long quaffed the nectar of defamation! When the "REPORT," which forms the text of this long article, was first published, we characterized it in terms at least as strong, as those which Sir James Scarlett has since employed.* Every syllable which we then put on pophetic record, has since been realized.

We may now *legally* say that the REPORT was a most *foul, false, and infamous calumny*. Mr. Wakley published that Report. If he did not see the *malus animus* with which it was indited, what are we to think of his head? If he did perceive the *malus animus*, and yet published the Report, what does he say to his heart? One of his own witnesses—the *Potatoe-merchant*—(and we verily believe him to be the *most honest* of all his witnesses, humble as was his origin)—characterized the Report of Sub-editor Lambert, (who modestly *swears* that he (Lambert) is a better surgeon than Mr. Cooper) as a "very *unprofessional Report*" !!!—We know not on which horn of this dilemma Mr. Wakley may choose to run—but between them he cannot steer, in the mind of any one, capable of reflection. This was the FIRST ACT of the DRAMA presented for his acceptance by the now celebrated manager of the *moral* department of the Lancet.† If an honourable man found that he had published an un-

---

* In the Commentary which we here append to this memorable trial, we beg it to be distinctly understood that, when we speak of the LANCET, or give it any character, we mean, unequivocally and conscientiously, a periodical WEEKLY JOURNAL, composed by various writers, to us totally unknown. By the term LANCET, therefore, we abjure and disclaim all or any personal allusion to editor, sub-editor, or individual writer—and for this plain reason, that we cannot tell who are the writers of articles that are anonymous. When we allude to the editor or sub-editor, we openly and candidly state them by name, and make ourselves responsible for our observations. This, we believe, is the honourable and honest path to pursue. We are sorry to say, that it is a path which Mr. Wakley himself has not taken, in his innumerable attacks upon us. He has, invariably, identified the Editor of this Journal with every writer in it—a conduct as unjust in a literary, as it is unmanly, in a personal point of view.—*Ed.*

† See Lambert's certificate of *moral* character, by which Clapham obtained his License from Apothecaries' Hall.

just stigma on the professional character of his neighbour, (and surely the conjoint letter of 150 students might have been sufficient evidence of this) what would naturally suggest itself as the remedy, and the measure to be pursued? Undoubtedly a direct and prompt acknowledgement of the error, and an expression of sorrow for what had occurred. Did Mr. Wakley offer this reparation for slandered fame and wounded feelings? No! he persevered in asserting that the *false* libel was a *true* statement! Thus ended the first act of the drama.

The second act was occupied in placing the plea of *justification* on the record. It was a natural consequence of the first act—and it greatly exasperated and empoisoned the sting of the libel. If a man, through heat of passion, or from inadvertency, publishes a calumny, and finds that he must take the legal consequences, he wisely determines not to aggravate the evil by any attempt to maintain the truth of that which is false. He accordingly pleads the GENERAL ISSUE, and thus takes the chance of mitigating the penalty incurred by himself, while he lessens the amount of evil sustained by the injured party. Did Mr. Wakley follow this rule? No. He deliberately pleaded a justification—and repeatedly published his determination to the world, thus tending, all in his power, to confirm the idea that Mr. Bransby Cooper was an ignorant surgeon, and unfit to perform an operation! When we consider the nature of the accusation—the terrible consequences of it to a medical man—and the laceration of feeling which it must produce in so many minds—we are utterly at a loss to know on what principle Mr. Wakley proceeded. If it was on a principle of JUSTICE, a jury of his country has proved *himself* to be unjust. If it was on any principle of humanity, philanthropy, science, or medical ethics, we leave it to his own professional brethren, in their moments of cool reflection, to pronounce on the wisdom, policy, or generosity of the measure.

This brings us to the THIRD ACT of the melancholy tragedy. For one medical man to publish a calumnious aspersion on the professional character of another, is bad—to put a justification on the record, is worse—but that the libeller should not relax his pursuit until he had *personally* poured forth the whole vial of his wrath in a crowded Court of Justice, expending every epithet of obloquy on the professional character of his victim, to that extent that the defamer sunk *exhausted* himself, and nearly expired in his terrible work of destruction—is almost incredible!! It is a phenomenon that was reserved for the nineteenth century!

The libeller, in this case, came into court in the character of a barrister—and he will, no doubt, be much gratified, when we say that he appeared at least as well qualified for that important function as for the less obtrusive station of SURGEON. He was attended by his FIDUS ACHATES, LAMBERT, who avowed himself, for the first time, as the writer of the libellous report, and the stipendiary sub-conductor of the LANCET! We shall defer our comments on the *latter*, for a few minutes, in order to make a reflexion or two on Counsellor Wakley. The propriety and the taste of a medical man coming into court to defend the truth of a libel on one of his own profession, we leave to the profession itself. The difficulties of medical practice were not sufficiently numerous—the prejudices of the non-professional public against the surgical practitioner were not sufficiently strong—the errors to which the most scientific surgeons are liable, were not patent enough to the world—till Mr. Wakley, that eminent and successful practical surgeon, of Bedford Square, thought fit to make an exposé of the ignorance of his brethren in the Court of King's Bench!! But, passing over Mr. Wakley's forensic qualifications, let us ask him, what the profession will think of the miserable attempt to horrify the minds of the court and the jury, by exhibiting a figure bound and pinioned as in the operation for lithotomy! A more disingenuous manœuvre never entered the mind of man. It was a preparatory step to the accusation, which he afterwards brought against Mr. Cooper, of violating the rights of a British subject—a "free agent," by persevering with the operation after the patient had cried out to be unbound! What Englishman will not blush for his country and his profession, when he reflects on this plea and this crimination brought forward in a court of justice by a man taking upon himself the censorship of the medical press! What man who has witnessed half a dozen operations, does not know that this exclamation for liberty is a very common occurrence, in most of the capital operations? The judge—who probably never saw a surgical instrument wet with blood, scouted the accusation, and absolved Mr. Cooper from any, the slightest blame on this occasion. Nothing can be more expressive of the character of the Lancet and its writers, than this fiery and unfair appeal to the passions of the populace—this sophistry of argument—this destitution of candour as well as knowledge.

We shall not dwell farther on Mr. Wakley's opening oration, which, like his concluding one, was but too familiar to the medical ear—in fact, a reiteration of the obsolete and disgusting harrangues in the Lancet, about hospital bats—corruptionists—public functionaries—freedom of the press, and all that kind of sickening slang. His speeches, on both days, were prominently distinguished by fluency of language—want of judgment—disregard of truth—and perfect freedom from all feeling of professional delicacy.

In his cross-examination of the witnesses, especially the plaintiff's witnesses—he evinced the most complete want of tact and skill. We appeal to every one present, whether the answers drawn forth did not invariably tell against himself. He wormed out not a single discrepancy of opinion among the mass of surgeons and physicians called into the witness-box—thus confirming, in a most extraordinary manner, the truth of their allegations. But he did more than this:—he displayed any thing but a practical acquaintance with the operation which he came into court to oppugn. His knowledge of anatomy was so exquisite, that he denied the possibility of a calculus being lodged above and behind the pubes—though the only witness, of any pretension to experience, who was called by himself, admitted such a circumstance—and even that it existed in the case in question.

Of his concluding harrangue, we shall here only notice one most remarkable example of his sophistry, disingenuousness, and want both of candour and judgment. He asked the jury whether, if they had the misfortune to be afflicted with calculus, they would, after what came out on the trial, select Mr. Bransby Cooper to perform the operation of lithotomy? If they would not, they must find a verdict for him!! Was there ever such a treacherous question proposed—or such a lame and impotent conclusion drawn from the implied answer! If a stranger to Mr. Cooper did not select him to operate, merely because the said Mr. Cooper was accused of operating unskilfully, the verdict must be given in favour of the libeller!! Is there a particle of sense, justice, or reason, in such a conclusion? No! The following is the fair question, and rational deduction from the answer. Would a surgeon who was in the habit of calling in Mr. Cooper to operate on his patients, cease to call him in, after seeing or hearing the whole case in question, *from an apprehension of his unskilfulness?* If the unfortunate case under litigation did not prejudice those who were the only proper judges of skill and dexterity, against the operator, then Mr. Wakley's ungenerous, disingenuous, and unprofessional question and conclusion fall to the ground. On this point he rested his cause so completely, that he triumphantly asserted the jury *must* find a verdict in his favour;—and yet they found a verdict against him.*

As to Mr. Wakley's batch of witnesses, whom he introduced as MAGNATES in the profession, and of the very first order of talent, skill, and experience, they made, with one or two exceptions, as contemptible a figure as ever was exhibited in a

---

* The world knows how Mr. Wakley boasted, a short time ago, that he would prove officially, on this trial, the exact circulation of the Lancet. Not a single attempt did he make when Lambert was in the witness-box, and, consequently, when he had a fair opportunity of "redeeming his pledge." No, no! He was wise enough not to touch on that point. True, his vanity permitted Sir James Scarlett to represent him as rolling about in his carriage, and earning five or six thousand a year!!! We venture to say, however, that Mr. Wakley keeps neither carriage, cab, nor shay—neither horse, mule, nor ASS—unless a literary HACK, near Clapham Common, can be included in this last denomination.

court of justice. Among the few who made any pretension to education, skill, or practical experience, there was an obvious prejudice or party-spirit—and, at all events, the most gross want of professional liberality—we had almost said of justice. The most honest, by far, of all Mr. Wakley's witnesses (Mr. Lee) declared, on oath, that no man but the operator himself could appreciate the difficulties of the operation. How, then, could Mr. Partridge, who sat behind the operator, reconcile his statement with any principle of professional feeling, liberality, etiquette—or honour? The harsh, uncharitable, UNCHRISTIAN judgment,* which Mr. Partridge passed on a brother practitioner, during this memorable trial, will one day be visited on himself! Both judge and jury negatived Mr. Partridge's FIAT—and we are convinced that the recollection of this trial, and of his own ungenerous, unprofessional evidence on it, will prove a source of bitter remorse to the latest day of his existence. It has been well observed by our contemporary, the Medical Gazette, that—"this man swore that he had never seen Wakley before; but kindred natures soon understand each other, and we observed that he sat by his new friend during the rest of the day, encouraging him by his presence, and assisting him with his advice." Verily, PARTRIDGE, thou hast paired with the VULTURE this time!

LAMBERT next figures on the scene! When the *report* of Mr. Cooper's case was first published, nine months ago, and when the reporter was totally unknown to us, we then said—*and we repeat it,* that—"the *anonymous* assassin of *character* may mix with the crowd, and plunge his concealed stiletto into the breast of his neighbour, unseen by mortal eye. But, when he lays his head on his pillow, a thorn will be there—and the voice of conscience will ultimately cry—

"Sleep no more, Macbeth has murdered sleep."

"Then will the pallid cheek and sunken eye betray the canker feeding on the soul."†

The above observations and reflexions were made in our indignation against the anonymous reporter of Mr. Cooper's case—and a jury of our country has proved the report to be false, scandalous, and malicious. We had no idea that any human being could have the hardihood to come forward, in a court of justice, and avow himself as the author of the said report. Yet Mr. Lambert has done so—and we envy him not the consequences. Mr. Wakley has shewn a deep insight into human nature, by an early selection of Mr. James Lambert for sub-editor of his journal. The man who could thrust his unhallowed finger into the DEAD, for the purpose, we will not say, of blasting the reputation of the LIVING, must be, of all others on the face of this beauteous globe, the fittest agent for weilding the LANCET!

"Heir to my virtues! man of equal mind!
"Skilled to condemn, as to traduce mankind,
"This steel receive! for thee reserved with care,
"To wield with judgment, and at last to bear."
*Byron.*

WE do not say that Mr. Lambert made a hole between the bladder and the rectum of the dead man. But Sir Jas. Scarlett has said so—and Sir James is an honourable man. The words of the honest quaker, too, must still vibrate in the ears of the public. LAMBERT. "Here is a hole between the bladder and rectum." HODGKIN. "Then, friend, thou hast made it thyself."

In coming into a court of justice—one as author, and the other as advocate, of the most atrocious calumny that ever issued from the press—Messrs. Lambert and Wakley appear to have made a bold experiment on the common sense of the profession, by trying how far impudent and unqualified assertion might stagger and beat down the modest and conscientious averments of TRUTH. On such an occasion, they, no doubt, judged it politic to touch the extreme goal—the ULTIMA THULÉ, of *possibility,* without entirely overleaping the utmost boundary of credence. They *did* approach that dangerous barrier!—but with an impetus that hurled them headlong, far, far beyond the mark! The Lancet has unconsciously passed the RUBICON of public feeling—it has drawn the sword against the dignity of the profession, and it may now fling away the scabbard! For it there is no retreat. Like Hannibal, it may, for a time, defend

* "Judge not, lest you be judged."

† See No. 17, p. 167.

itself in its Appenine fastnesses—but

"Nor Alp nor ocean shall it pass again."

No journal ever took a wider range for the theatre of its defamations than the Lancet. It appeared to think the sphere of its circulation and abuse almost illimitable—and that no surgeon would have the courage to face a defamer of his surgical skill before a public tribunal—especially when a JUSTIFICATION was put on the record. Medical society is deeply indebted to Mr. Bransby Cooper for braving, at once, the literary ruffians (and they are many) of his own profession, and the censorious gossips of the world at large. He has been the instrument of exposing the secret springs and agencies of the most abominable journal that ever disgraced the medical profession:—

"A coward brood which mangle as they prey

"By hellish instinct, all that cross their way;—

"Aged or young—the living or the dead,

"No mercy find—these harpies must be fed."

*Byron.*

The judge's reprimand will ring for ever in Lambert's ears. Towards the close, Lord Tenterden reproved him, observing, that "*he had not answered one question in a straight-forward manner like a man.*"

Such is the SUB-EDITOR of the LANCET! We know not which to scorn most—the hireling employed by such a publication —or the journal *obliged* to employ such a hireling! Here Wakley is between the horns of another dilemma! If he keeps Lambert as SUB-EDITOR of the LANCET, it is not difficult to foresee the result. If he quarrels with him, the "secrets of the prison-house" will come out—and then————! In short, the predicament in which the editor and his SUB now stand, may be more easily imagined than described! Such is the state of the medical FREE PRESS, as it is called, in this country—free only from all shackles of literary justice—a slave to the worst of passions in its anonymous contributors!

A reporter, perhaps the enemy of an able and amiable surgeon, writes a most malicious and scandalous libel on his professional character, dressed up in the most sickening and "unprofessional" language. The "free press" receives it. Its editor mourns over the destruction which the libel may cause to an innocent and unoffending man, the father of a large family dependent on him, and he on his reputation for bread! But what of all this? Think on my sacred office. "My reporter has furnished me with a paper—and I am in duty bound to publish it!!" What a Lambert has written, a Wakley *must* print! But this is not all. A fictitious tragedy is got up in a public institution, (Glasgow), and the medical officers are sacrificed en masse, to save the trouble of individual literary execution. The tragedy is sent under a fictitious signature to the FREE PRESS—the Editor examines the document—*suspects* that it is fictitious—but remembering the sanctity of his high office, finds himself *in duty bound* to publish the flagitious calumny!

Mr. CLAPHAM, aged 20 years last January, who practises as a surgeon with his father, was another of the talented and experienced practitioners brought into court by Wakley. He sat on the third bench—heard the sound strike the stone —swore that the various instruments were used "*out* of their accustomed order" (though Mr. Partridge swore that they *were used in* the accustomed order)—and, in short, entirely disapproved of Mr. Bransby Cooper as an operator! When cross-questioned by Sir James Scarlett respecting his age, it came out that he had either sworn a false oath, or forged a false certificate! He was ordered out of the witness-box by the judge—and he is now, we understand, under indictment by the Apothecaries' Company! This Clapham is a cousin of Lambert's—and Lambert swore, that "he did not assist in procuring his license." Yet, on the evening of the second day of the trial, Mr. Lambert's certificate to the *moral* character, &c. of Mr. Clapham, was produced in court—by which certificate the said Clapham was enabled to apply for his license! How Mr. Lambert can reconcile these facts it is difficult to imagine. Perhaps Mr. Clapham, when he applied to Mr. Lambert for a certificate of his *moral* character, concealed the purport of it, and only wished to have in his possession a document that would be a "feather in his cap" all his life afterwards—and which is no doubt encircled with a broad gilt frame, and conspicuously hung up in his father's shop!!

We might here pause for a moment, and contemplate the examples of *moral retribution* which are every day brought to light by the workings of inscrutable Providence! A foul and libellous charge is made against an excellent, amiable, and expert surgeon, for the purpose of ruining his character, and blasting his prospects in life. The charge is falsified —and destruction is brought—not on one or two, but on several of the agents! We say nothing of Wakley and Lambert. But see what Mr. Clapham has brought himself to by espousing their bad cause! The grey hairs of his parent will be brought prematurely and in sorrow to the tomb—and the venerable Lord Tenterden's order for him to quit the witness-box, is a stigma, which the whole age of man can never efface! Such are the glorious fruits which result from the medical youth of England imbibing the principles of the LANCET!! The fathers and the masters of young men have here an example of the fatal security in which they thought themselves placed, when reading scandalous attacks on their neighbours! The evil will come home to their own bosoms in the end! The senior and junior Claphams, being staunch supporters and ardent admirers of the LANCET, enjoyed, no doubt, every Saturday, whole lots of laughter at the expense of the "old hags of Rhubarb Hall," and other such monstrous witty sayings and doings in their favourite JOURNAL! We rather suspect that, by this time, their merriment has taken a very different turn!

JOACHIM GILBERT, the man of delicate nerve and tender feelings—who was mistaken by Sir James Scarlett for an hostler—and who ran away in the middle of the operation, did not decamp before he furnished himself with a good stock of charitable criticisms on the operator. Mr. Cooper, in Gilbert's opinion, "held his arm too high"—"used great violence"—"twisted his fingers about in the wound"—asserted, (on his oath, of course) that the second incision "did *not* reach the bladder"—but "went between the rectum and bladder" (!!!)—Swore that "there was no flow of urine"—that Mr. Cooper thrust in the forceps "as if he meant to *stab the man*," &c. &c. This Gilbert is assistant to Mr. Wakley's brother-in-law, Mr. Phelps.—We did not happen to see this witness, and therefore cannot speak as to the "hard features" described by Sir James Scarlett. From the hard words, ready oaths, and sweeping censures, in which he dealt, we have no doubt of his family likeness to the other members of the house of Wakley.

> "Facies non omnibus una,
> "*Nec diversa tamen.*"

This witness crowned the matchless absurdity of his evidence, by swearing (for all evidence is on oath) that, in his opinion, "a very ignorant surgeon might, BY ACCIDENT, tie the subclavian artery, with success." If twenty Munchaussens were again to visit this world, they would not be able to invent so monstrous and absurd a question as that which Wakley proposed, and Gilbert answered! *

The other witnesses for the defendant, which completed this motley group, need no farther notice:—

> "An undistinguished crew,
> "O'er whom her darkest shade Oblivion threw."

"PUPILS, (as a cotemporary observes) forgetful of the modesty that becomes their age — a DEMONSTRATOR of two months standing to an itinerant lecturer (which Demonstrator swore that he did not know the name of Wakley, to whom he was in the habit of sending communications!)—a hireling slanderer—a potatoe-merchant (the most honest and honorable witness among them)—and—a PERJURER"!! †

The witnesses that came forward to bear testimony to Mr. Cooper's skill, formed a remarkable contrast with the

---

* Mr. Wakley, the defendant, and the ADVOCATE of his own cause, drew his own bewildered witness into this exposure. Would any man, who possessed a particle of sense, or the slightest knowledge of operative surgery, think of asking a witness "whether an ignorant surgeon might not tie the subclavian artery *by accident*, with success? Yet, such a question was asked by Advocate Wakley, and answered in the affirmative by his witness Gilbert!! We venture to surmise that, if ever, either Wakley or his witness ties the subclavian artery—it will be "BY ACCIDENT." The man that asked this question is he who sets himself up as the patron and representative of the GENERAL PRACTITIONERS of England!

† Med. Gaz. Dec. 20, 1828. We believe the term *perjurer* is improper—he was only a *forger*.

insolent, impudent, illiberal, and uncharitable band that preceded them. As far as operative surgery is concerned, we might have been justified in adding the epithet *ignorant*—with the exception of one individual—but we shall not be so uncharitable as the unenviable censors of Mr. Cooper's conduct on this occasion. The evidence of Mr. Callaway was *instar omnium*. With that inconsistency inseparable from a bad cause, the LANCET first represented Mr. C. as a better surgeon than Mr. Cooper—then as an injured man, over whose head Mr. Cooper was placed — as the very best judge of the steps of the operation—and, when his testimony went against it—as favouring his successful rival, from sordid and interested motives ! ! Such are the miserable shifts to which libellous aspersers of character are reduced. Mr. Callaway, who held the staff, and assisted at the operation, gave his most unqualified testimony to the skill, dexterity, and coolness of the operator, on this, as well as on many other occasions.

Mr. Callaway's cross-examination elicited some circumstances that are highly deserving of notice. Every body knows how President Haslam has been bespattering, with fulsome praise, the Editor, and Sub-Editor of the FREE-PRESS of late; and how much the Bolt-court Society is fallen in the estimation of the respectable members of the profession. Wakley asks Mr. Callaway whether he did not call Mr. Cooper an idiot, at the Kent Medical Dinner? No, says Mr. Callaway, *your friend, Dr. Haslam*, questioned me, and, therefore, I was on my guard! This speaks volumes. Our cotemporary, of the Gazette, has made some comments on this part of the evidence, which we recommend to our readers.* Let the Bolt-Court Society look to this! Let the council who have been browbeaten and impugned by Haslam, bestir themselves, and maintain their dignity. It would appear, that the "jackal," (for all jackal's have keen scent) begins to smell a rat—if not something worse—and has been brought to a negation of Wakley's insidious question, by Mr. Callaway himself, about the *idiocy* of Mr. Cooper. Here, then, is another intestine division in the house of Wakley—and among the friends of the FREE-PRESS! Mr. W. asked Mr. Callaway the above question—and, therefore, he either *invented* the grounds for the question, or has been misled by some of his jackals! Utrum horum, &c.?

The host of eminent surgeons who gave testimony to Mr. Cooper's talents and dexterity may be passed over, as not a single discrepancy could be extracted from them. Every cross-question by Wakley tended to injure his own cause. Sir Astley Cooper frequently set the Court in a roar, by the ineffable contempt with which he treated the LIBELLER—and all with the greatest good humour.

Having placed a full, though very inadequate account of Sir James Scarlett's speech before our readers, we can say nothing capable of enhancing its eloquence and force. It will stand among the most splendid efforts of forensic power yet on record. In many parts, it was sublime, even to the terrific! The character which he drew of the Lancet, its editor, and its sub-editor, can never be forgotten by the audience, or wiped off by the parties. Their condition, if they had any feeling, was only to be envied by the slave descending for the first and also for the last time into the Peruvian mine—never more to see the light of Heaven! Golconda's gems could not tempt us to go through the ordeal of that day! None but a salamander could have withstood the fiery torrent of indignant denunciation that flowed from the plaintiff's counsel on this memorable occasion! When Sir James came to that part of the "drama," in which Lambert is accused of pushing his unhallowed finger through the dead man's entrails, in order (as Sir James averred) to furnish a proof of his victim's misconduct, the blood must have curdled in the veins of every human being within the walls of the Court! To be pointed out as the author of such a transaction—

"*Digito* monstrari et dicier HIC EST,"

was, indeed, one of the most awful examples of retributive justice that ever was witnessed on earth. It furnished a moral lesson that should never be forgotten. The ferocious pleasure apparent in every line of the libellous and malignant report of the case, has been dearly earned and severely punished by the tortures of

* Med. Gazette, Dec. 20, 1828, p. 101.

this trial, independent of what is to come!

We have now but one or two more topics to advert to, before we close this commentary. One universal sense of surprise has been evinced at the smallness of the damages—the general anticipation having been that 500 pounds, at least, would have been awarded. Although this very surprise tells against Wakley's cause, in the most unequivocal manner, yet, with his usual jesuitical sophistry, he endeavours to turn the award of low damages into a proof of triumph on his part. He has been rather cautious, however, in urging this point, aware of the *argumentum ad hominem* which he knew WE could and would bring effectually to bear upon it.

When Mr. Wakley brought his celebrated action against ourselves, and when the sting of the libel was a charge of felony, what damages were awarded?—Precisely the same sum that a jury awarded to Mr. Cooper for the charge of unskilfulness. Let Mr. W. reflect on this parallel, and be silent about the amount of damages.

We shall say a few words, however, on this subject. The general but erroneous impression on the public mind is, that the damages, in a case of libel, are awarded as a *punishment* for the offence. This is not the case. The term as well as the idea of *punishment*, in a civil action, is misplaced. Punishment belongs to a criminal process, and comes in the shape of imprisonment or fine. But damages in a civil action, designate the *compensation* for such injury as a jury thinks the plaintiff has sustained by the false accusation. The impossibility of a jury being able to form a proper estimate of the injury, on all occasions, is the cause of the inadequate or inordinate sums we sometimes see awarded. Thus in the first of the two cases in point, the jury determined that Mr. Wakley's character had sustained damage to the amount of one hundred pounds, by the charge of ARSON—and in the second case, a jury came to the conclusion that Mr. Bransby Cooper's character sustained damage to the extent of 100 pounds, by the accusation of unskilfulness. This is the true state of the case. If there be any thing like *punishment* in a civil cause, it is the payment of costs of suit, which the defendant has to defray *on both sides*, when he fails in his defence. The VERDICT, therefore, is the criterion of the truth or falsehood of the libel—the damages are given for the supposed injury received—and the costs of suit may be considered as the punishment for failing in a bad cause. The jury being aware that the expenses of these libel actions are tremendous, take that point into consideration, in estimating the damages. The recent action will not cost Mr. Wakley a farthing less than ONE THOUSAND GUINEAS.

The last topic which we shall touch upon, is, the moral effect of this transaction—or, the probable consequences which may result from it, not only to the parties concerned, but to the whole profession.

We are among those who believe, (because we have lived long enough to see numerous proofs) that evil ultimately corrects itself, even in this world, and that INJUSTICE, however successful for a time, comes in for its award in the end. In the drama of defamation which has been enacting during the last few years, it was difficult not to foresee that every incident tended, directly or indirectly, towards the completion of the catastrophe. Every time that a man's fame was traduced with impunity, the libellers became more bold—till, at length, they considered themselves invulnerable. The last and most cruel of these literary assassinations proves that nothing like fear could have operated on the minds of the dramatis personæ. This boldness was engendered by the supineness of the profession, and by the culpable taste for purchasing and perusing a neighbour's scandal. The impolicy of this propensity is now made amply patent; but not before an honest and honorable man has been nearly sacrificed. Mr. Bransby Cooper is a victim to the national appetite for slander! For although he is honourably acquitted by every competent judge in the profession; yet has he suffered and will suffer from the prejudice engendered in ignorant minds by this wanton attack on his professional reputation. A surgeon's skill, like a soldier's courage, or a woman's virtue, cannot be questioned without detriment—and, therefore, Mr. Cooper's acquittal and triumph constitute no adequate compensation for the injury he has sustained, and will, for some time sustain, in extra-professional society. The medical profession then owe it to themselves—to an injured member of their body—to justice, and honour—to make reparation, as far as in them lies, for an injury to which they are in some degree accessary, by their tolerance of the libellous publication. It is for them to mark their indignation, in every possible way, at the calumny—and to foster and protect the calumniated—falsely calumniated victim of systematic defamation.

Already the impulse has been given—and the generous youths who frequent the Borough hospitals, have unanimously expressed their detestation of the libel, and admiration of their teacher.* Already has the Westminster Medical Society taken measures for the expulsion of Lambert—for who would remain members of a medical society to associate with the self-acknowledged reporter of Mr. Cooper's case?† A special general meeting of the Physical Society of Guy's Hospital is called—and the purpose is sufficiently obvious. These are omens of a more auspicious æra—and we trust that meetings and resolutions will take place in every town in England, to express to Mr. Bransby Cooper, the horror which the respectable orders of society entertain for the libel on his character, and their admiration of his courage, in dragging the offenders into open day. By this procedure, and this procedure only, can the profession hope to wipe off a portion of the stains to which it has passively submitted for the last five years.

Pudet hæc opprobria nobis
Et potuisse dici, et non potuisse refelli.

---

* The following is the resolution of the medical students.

"GUY'S HOSPITAL.

"At a Meeting of the Pupils held at the Anatomical Theatre of Guy's Hospital, on Monday, Dec. 15, 1828,

"It was resolved unanimously—That in consequence of the manly and upright manner in which their valuable and inestimable teacher Mr. BRANSBY COOPER has come forward to vindicate his character against one of the most foul, unmerited, and wicked attacks that malice ever devised for the ruin of professional reputation, the Pupils feel it their duty not only to offer him their warmest congratulations upon the triumphant result of the late trial in the Court of King's Bench, but to present him with a lasting testimonial of their gratitude towards him as their teacher, and as a token of the high sense which they entertain of his skill as an operator, of his judgment as a surgeon, and of his character as a man.

"It was resolved unanimously—That this object be carried into effect by presenting Mr. BRANSBY COOPER with a piece of plate, bearing an appropriate inscription, the expenses of it being defrayed by a subscription, to be confined to those gentlemen who have been entered as pupils of St. Thomas's and Guy's Hospitals, and each contribution to be limited to the sum of half a guinea.

"It was resolved unanimously—That subscriptions continue to be received by one of the Committee at Guy's Hospital, for one month from the present date."

† One of the most talented and honorable presidents of the Society has publickly expressed his determination to withdraw from the Society, if Lambert remains. The example will be followed by every respectable member of the institution.

P.S. The meeting at the Freemason's Tavern, on Tuesday night, the 23d December, advertised for the triple purpose of "SURGICAL REFORM"—"Support of the FREE-PRESS"—and a "subscription for Wakley"—will probably open the eyes of the radical levellers in this country. Not a single medical man, of the slightest pretension to notoriety, took part in the debate—with the exception of Dr. SHEIL, who ridiculed and exposed the whole proceeding! It will convulse the profession with laughter, when we inform them that the CHAIR OF SURGICAL REFORM was filled, on this occasion—not by Mr. LAWRENCE—but by a Mr. PATY, of heaven knows where—and that the principal supporter of the meeting was a Mr. HENSLEIGH, practising (we have no doubt with great credit in his way) near the YORKSHIRE STINGO, Paddington! Risum teneatis amici?—There was no subscription for the hero of the FREE PRESS—but a dinner was determined on, at some future opportunity, if money could be raised for that purpose. There was great difficulty in procuring STEWARDS—and there will be still more, we apprehend, in finding COOKS! To dinner with what appetite ye may!

